# Macrophage depletion through colony stimulating factor 1 receptor pathway blockade overcomes adaptive resistance to anti-VEGF therapy

**DOI:** 10.18632/oncotarget.20410

**Published:** 2017-08-24

**Authors:** Yasmin A. Lyons, Sunila Pradeep, Sherry Y. Wu, Monika Haemmerle, Jean M. Hansen, Michael J. Wagner, Alejandro Villar-Prados, Archana S. Nagaraja, Robert L. Dood, Rebecca A. Previs, Wei Hu, Yang Zhao, Duncan H. Mak, Zhilan Xiao, Brenda D. Melendez, Gregory A. Lizee, Imelda Mercado-Uribe, Keith A. Baggerly, Patrick Hwu, Jinsong Liu, Willem W. Overwijk, Robert L. Coleman, Anil K. Sood

**Affiliations:** ^1^ Departments of Gynecologic Oncology and Reproductive Medicine, The University of Texas MD Anderson Cancer Center, Houston, TX, USA; ^2^ Center for RNAi and Non-Coding RNA, The University of Texas MD Anderson Cancer Center, Houston, TX, USA; ^3^ Department of Cancer Biology, The University of Texas MD Anderson Cancer Center, Houston, TX, USA; ^4^ Department of Bioinformatics and Computational Biology, Division of Quantitative Sciences, The University of Texas MD Anderson Cancer Center, Houston, TX, USA; ^5^ Department of Melanoma Medical Oncology-Research, Division of Cancer Medicine, The University of Texas MD Anderson Cancer Center, Houston, TX, USA; ^6^ Department of Pathology, Division of Pathology and Laboratory Medicine, Section of Gynecologic Pathology, The University of Texas MD Anderson Cancer Center, Houston, TX, USA; ^7^ Department of Leukemia, The University of Texas MD Anderson Cancer Center, Houston, TX, USA

**Keywords:** adaptive resistance, anti-VEGF therapy, tumor associated macrophages, tumor microenvironment, CSF1R inhibition

## Abstract

Anti-angiogenesis therapy has shown clinical benefit in patients with high-grade serous ovarian cancer (HGSC), but adaptive resistance rapidly emerges. Thus, approaches to overcome such resistance are needed. We developed the setting of adaptive resistance to anti-VEGF therapy, and performed a series of *in vivo* experiments in both immune competent and nude mouse models. Given the pro-angiogenic properties of tumor-associated macrophages (TAMs) and the dominant role of CSF1R in macrophage function, we added CSF1R inhibitors following emergence of adaptive resistance to anti-VEGF antibody. Mice treated with a CSF1R inhibitor (AC708) after anti-VEGF antibody resistance had little to no measurable tumor burden upon completion of the experiment while those that did not receive a CSF1R inhibitor still had abundant tumor. To mimic clinically used regimens, mice were also treated with anti-VEGF antibody and paclitaxel until resistance emerged, and then a CSF1R inhibitor was added. The addition of a CSF1R inhibitor restored response to anti-angiogenesis therapy, resulting in 83% lower tumor burden compared to treatment with anti-VEGF antibody and paclitaxel alone. Collectively, our data demonstrate that the addition of a CSF1R inhibitor to anti-VEGF therapy and taxane chemotherapy results in robust anti-tumor effects.

## INTRODUCTION

Angiogenesis and VEGF are known to play an important role in the progression of ovarian and other cancers, making bevacizumab an important drug in standard treatment regimens [1]. Even though bevacizumab provides modest increases in survival, patients eventually develop progressive disease and relapse despite continuous anti-VEGF therapy [2]. Of the proposed mechanisms of anti-angiogenic therapy resistance, here we focus specifically on the role of macrophages [3]. The anti-angiogenic environment creates hypoxia that recruits TAMs and other myeloid cells to the tumor microenvironment, promoting angiogenesis *via* the secretion of angiogenic molecules such as FGF-1/2, MMP9, and Ang2 [4]. Therapy that overcomes adaptive resistance to anti-VEGF therapy by targeting macrophages could potentially improve clinical outcomes of cancer patients.

Although many pathways exist to deplete macrophages, we focused on the CSF1R pathway for its role as the primary receptor for macrophage survival, and its low side effect profile [5]. CSF1R inhibition is best described in clinical trials for diffuse-type tenosynovial giant cell tumor (dt-GCT), a disease characterized by overexpression of CSF1 [5]. Administration of emactuzumab, a human monoclonal IgG1 antibody against CSF1R, led to objective response in 86% of patients [5]. 78% of patients with evaluable tumor samples showed a significant reduction in CD68^+^/CD163^+^ and CSF1R^+^ macrophages [5].

In the present study, we used two CSF1R inhibitors with different mechanisms of action to determine the effects of CSF1R inhibition in combination with anti-VEGF therapy in the setting of adaptive resistance in ovarian cancer models. We also evaluated pathways affected after treatment with a CSF1R inhibitor in the setting of adaptive resistance to anti-VEGF therapy, by performing full immune profiling with cytometry by time-of-flight (CyTOF).

## RESULTS

### Anti-tumor effects of targeting TAMs in ovarian cancer models

**Figure 1:**
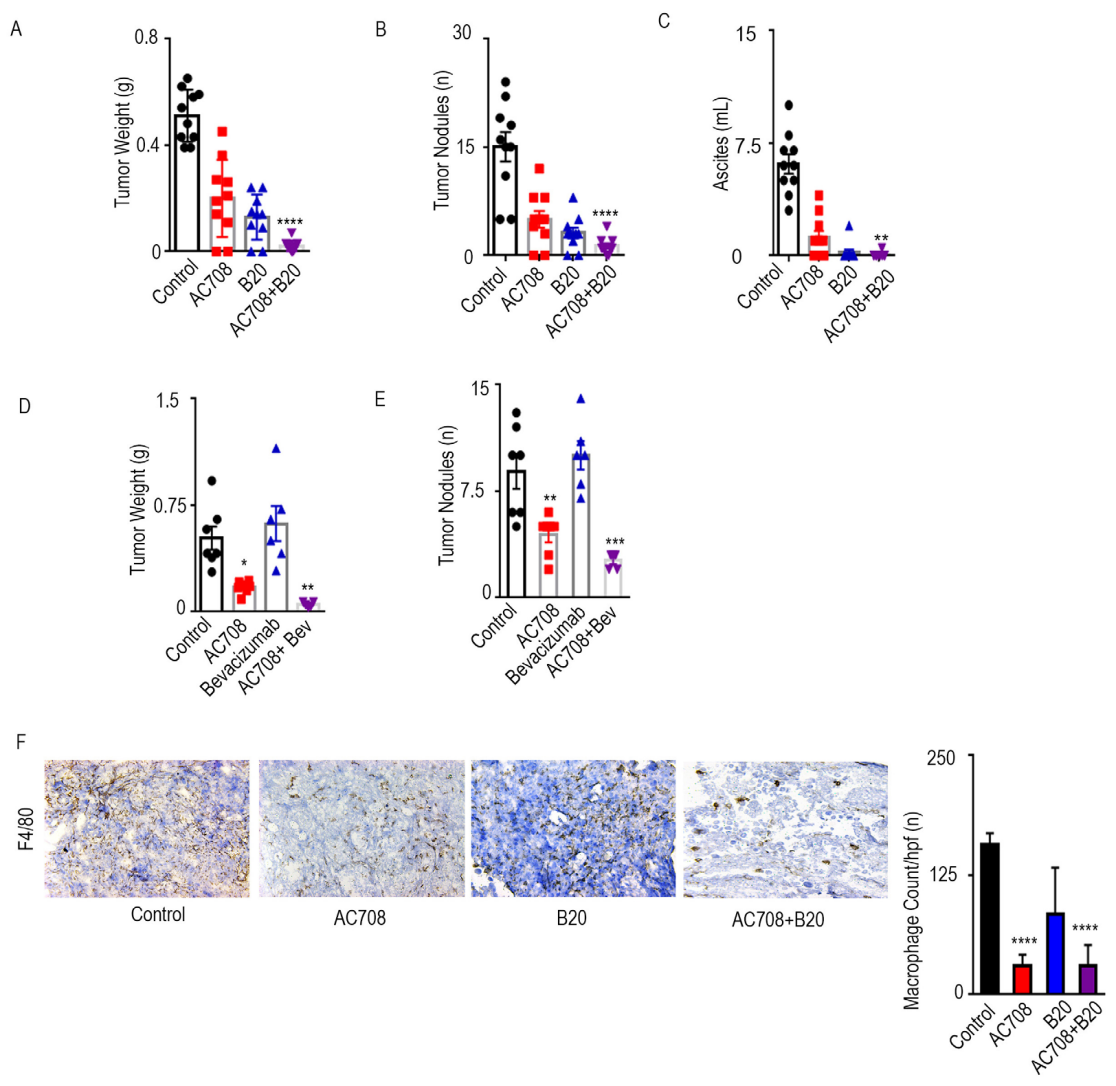
AC708 combined with B20 decreases tumor burden in syngeneic and PDX mouse models C57Bl/6 mice received IG10 murine ovarian cancer cells by intraperitoneal injection and were randomly assigned to treatment with AC708, B20, or the combination. Bar graphs show the tumor weight, tumor nodules, and volume of ascites **A.**-**C.** NOD-SCID mice were injected intraperitoneally with ascites from a patient with HGSC and randomly assigned to treatment with AC708, bevacizumab, or the combination. Tumor weight and number of nodules are shown **D.**-**E.** IG10 tumor samples from all groups were stained for F4/80 and total macrophage count was compared between groups **F.**. The bar graph represents mean number of macrophages from 5 randomly selected high power fields at 20x high power. * denotes p≤0.05, ** denotes p≤0.01, *** denotes p≤0.001, and **** denotes p≤0.0001.

First, we evaluated the effect of the CSF1R inhibitor, AC708, on tumor burden in the IG10 syngeneic mouse model. AC708 (also known as PLX73086), is a small molecule CSF1R inhibitor with significant specificity for CSF1R that is currently being tested in clinical trials [6]. C57/Bl6 mice were inoculated with IG10 cancer cells *via* intraperitoneal route. Given the strong pro-angiogenic role of TAMs, we first tested the effects of AC708 in combination with the B20 anti-VEGF antibody (targets both mouse and human VEGF). Mice were randomized into 4 groups: 1) control, 2) AC708, 3) B20, 4) AC708 + B20. B20 treatment alone resulted in a 75% decrease in tumor weight, but the combination of AC708 and B20 led to a 96% decrease in tumor weight, as well as significant decreases in tumor nodules and ascites compared to control (Figure 1A-1C). Representative pictures of tumor burden in each group are shown in Supp Figure 1A.

We next utilized a PDX model established from a patient with HGSC at our institution. The mice were injected with ascites from the PDX model and treatment began approximately 4 weeks after tumor cell inoculation, using the same groups as the experiment above. Tumor weight (Figure 1D) and nodules (Figure 1E) were decreased by 67 and 50%, respectively, in the group treated with AC708 *versus* control. The effect, however, was much greater in the group treated with AC708 and B20 compared to control, yielding a decrease in tumor weight and tumor nodules of 90 and 71%, respectively (p ≤ 0.01). The AC708 plus B20 group had no ascites present upon necropsy (Supplementary Figure 1B).

Next, we examined the effect of AC708 on macrophage count among the treatment groups for the syngeneic mouse model. AC708 decreased macrophage content in tumors by 81% both when compared to control, and when combined with B20 (p ≤ 0.0001), as confirmed by immunohistochemical staining with F4/80 (Figure 1F). Similar effects were found in the PDX model (Supplementary Figure 1C).

### CSF1R inhibitor restores sensitivity to anti-VEGF therapy and depletes macrophages in the setting of adaptive resistance

**Figure 2:**
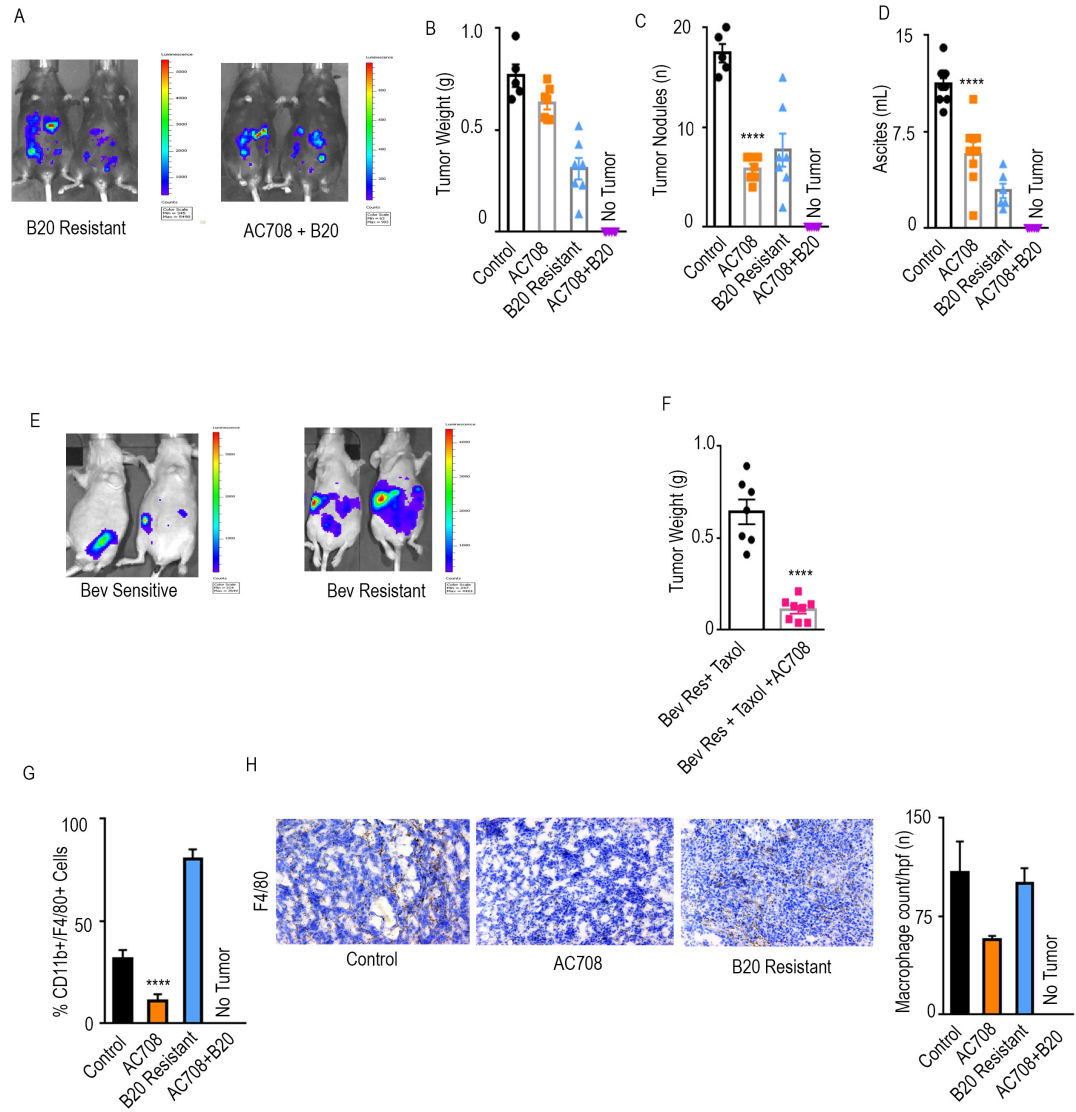
AC708 reduces tumor burden in setting of adaptive resistance to anti-VEGF therapy Bioluminescent signal differences between B20 resistant mice with and without the addition of AC708 are shown. Automatic exposure time was used **A.**. Tumor weight, tumor nodules, and volume of ascites **B.**-**D.** are shown in IG10 murine ovarian cancer model of adaptive resistance treated with AC708, B20, or the combination, after resistance was determined by bioluminescent imaging. **E.** demonstrates bioluminescence imaging differences in those mice sensitive *versus* resistant to treatment with bevacizumab and paclitaxel in OVCAR432 HGSC model. Automatic exposure was time was used. Tumor weight of the OVCAR432 model shown in groups resistant to bevacizumab, plus paclitaxel, with and without AC708 **F.**. **G.** Quantification of macrophages from IG10 model treated with AC708, B20, or the combination in the setting of adaptive resistance. Macrophage content was determined by the percentage of CD11b^+^/F4/80^+^ cells out of CD45^+^ cells, using flow cytometry. The same groups were also stained for F4/80 *via* immunohistochemistry to quantify macrophages **H.**. **** denotes p≤0.0001.

Next, we sought to evaluate the consequences of CSF1R inhibition in the setting of adaptive resistance to anti-VEGF therapy. TAMs contribute to adaptive resistance *via* production of pro-angiogenic cytokines upon recruitment from the bone marrow under hypoxic conditions [3]. C57/Bl6 mice were inoculated with IG10 luciferase-labeled (IG10-Luc) ovarian cancer cells intraperitoneally and then randomized to 3 treatment groups: 1) control, 2) B20, and 3) AC708, with treatment beginning on day 21. The mice were imaged *via in vivo* imaging systems (IVIS) weekly to determine response to treatment with B20 based on bioluminescence signal (Figure 2A). Mice determined to be sensitive to B20, as defined by initial decrease and then plateau in luminescence signal, were sacrificed approximately one week after sensitivity was determined. The mice determined to be resistant to treatment, defined as an initial decrease and then steady increase in luminescence signal, either continued with B20 alone or received AC708 in addition. Tumor nodules and ascites were decreased by 66 and 49% in the AC708 group as compared to control, while tumor weight decreased by 18% (Figure 2B-2D). The group that received AC708 at the point of B20 resistance had almost a complete response, leaving very little tumor tissue for analysis, compared to the group that continued on B20 alone at the point of resistance. The volume of ascites in the group treated with B20 plus AC708 was minimal.

Next, we performed an experiment designed to mimic the clinically used regimen of bevacizumab and paclitaxel, to determine the effects of CSF1R inhibition in the setting of adaptive resistance to anti-VEGF therapy with the addition of chemotherapy [2]. Mice were inoculated with OVCA432 ovarian cancer cells intraperitoneally and treated with bevacizumab and paclitaxel starting on day 21. IVIS imaging was performed weekly and mice were separated into those sensitive and those resistant to treatment (Figure 2E). Sensitive mice were sacrificed soon after the mice were divided, while those determined to be resistant received AC708 in addition to their current regimen. Tumor weight, tumor nodules, and ascites (Figure 2F, Supplementary Figure 2A-2B) were significantly decreased by 83, 84, and 98%, respectively, in the group that received AC708 at the point of bevacizumab resistance, as compared to mice that continued on bevacizumab and paclitaxel only (p ≤ 0.05).

Macrophage counts in the IG10 model were significantly decreased (*p* < 0.001) in the group that received AC708, *versus* the B20 resistant group, as determined by CD11b^+^/F4/80^+^ cells of the CD45^+^ population on flow cytometry (Figure 2G). A 65% decrease in macrophages was found in the AC708 group *versus* the control group, while an 86% decrease was seen in the AC708 group *versus* the B20 resistant group. F4/80 staining *via* immunohistochemistry (Figure 2H) revealed a 47 and 43% decrease in number of macrophages in the AC708 treated group as compared to control and the B20 resistant group, respectively. Given the complete response seen in the group that received AC708 after B20 resistance, we were unable to process any measurable tissue for analysis of macrophage content. A 71% decrease in macrophage count was seen in the group treated with the combination of bevacizumab, paclitaxel, and AC708 at the point of bevacizumab resistance, as compared to the group resistant to bevacizumab and paclitaxel (Supplementary Figure 2C).

### Molecular pathways affected by macrophage depletion

**Figure 3:**
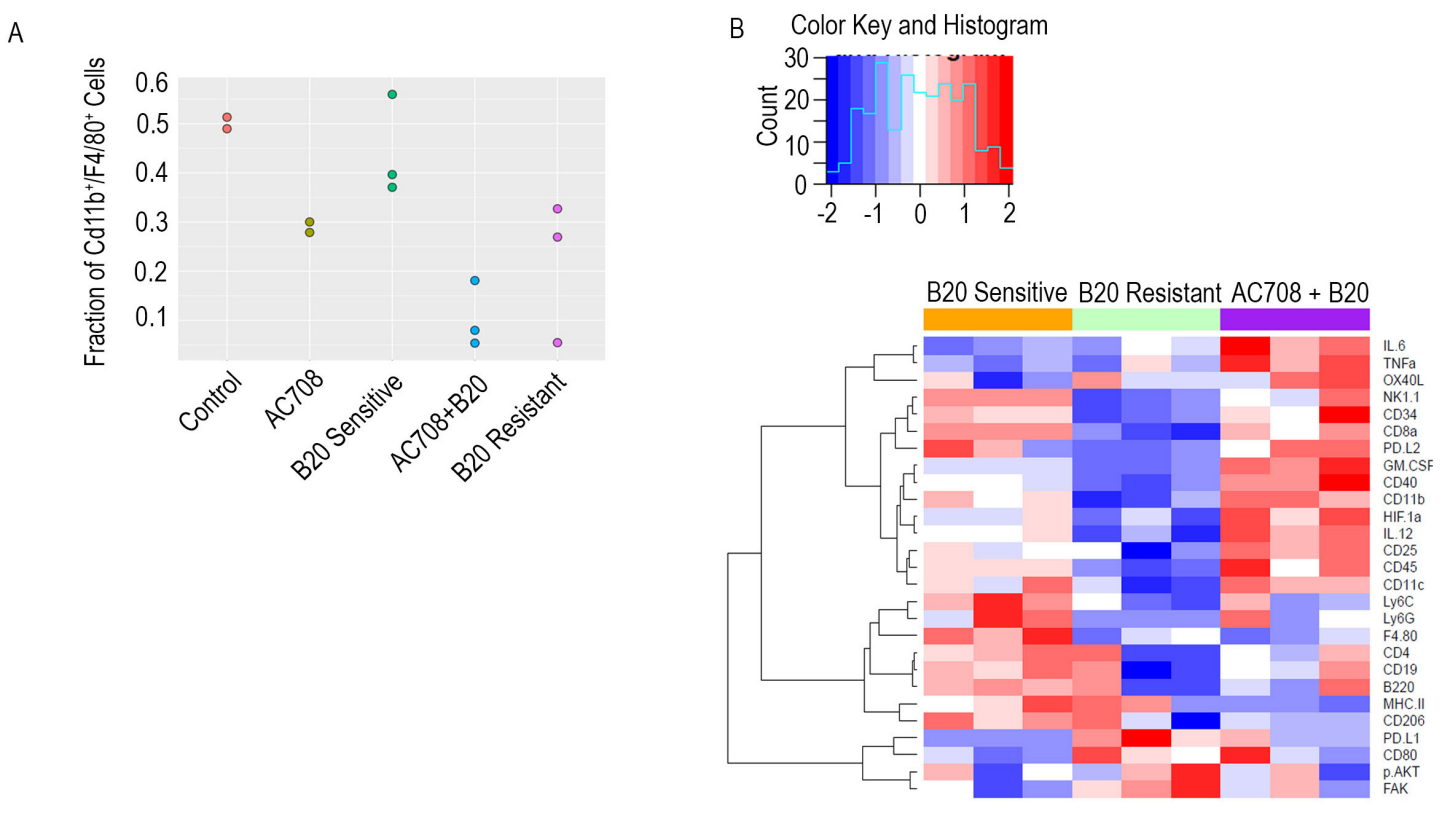
Macrophage depletion in the setting of adaptive resistance is confirmed with CYTOF Immune profiling *via* CyTOF was performed on IG10 tumor tissue harvested and processed from mice treated with AC708, B20, or the combination in the setting of adaptive resistance to B20. **A.** demonstrates the fraction of macrophages among groups determined by Cd11b^+^/F4/80^+^ cells out of CD45^+^ cells using CyTOF. Expression of the myeloid markers in the macrophage populations of each group are shown in the heatmap **B.**. Pathways involving PD-L1, p-AKT, and FAK were upregulated in the B20 resistant treated samples, and conversely decreased in the B20 sensitive and combination groups.

To understand the potential mechanisms underlying the response to therapy, we next set up another adaptive resistance model using the IG10 cell line in order to proceed with comprehensive immune profiling. At the completion of the adaptive resistance experiment in the syngeneic mouse model, immune cells were isolated from fresh tumor tissue from 5 groups: 1) control, 2) B20 sensitive, 3) AC708, 4) B20 resistant, 5) B20 resistant + AC708. The tissue was processed, stained, and subjected to profiling using cytometry by time-of-flight (intracellular and surface markers are listed in Supplementary Table 1). Compared to control samples and those determined to be resistant to B20, tumors from the AC708-only treated mice and the B20 resistant plus AC708 treated tumors had decreased macrophages, as determined by the fraction of CD11b^+^/F4/80^+^ cells in all CD45^+^ live cells (Figure 3A). Both groups treated with AC708 showed a significant decrease in macrophages when compared to the untreated groups (p ≤ 0.05). Figure 3B represents the expression profile of all the myeloid markers in the macrophage population across all groups. PD-L1, p-AKT, and FAK expression were elevated in samples resistant to B20, while the expression of these markers was decreased once AC708 was added to B20 resistant mice.

### Anti-tumor effects of additional CSF1R inhibitors

**Figure 4:**
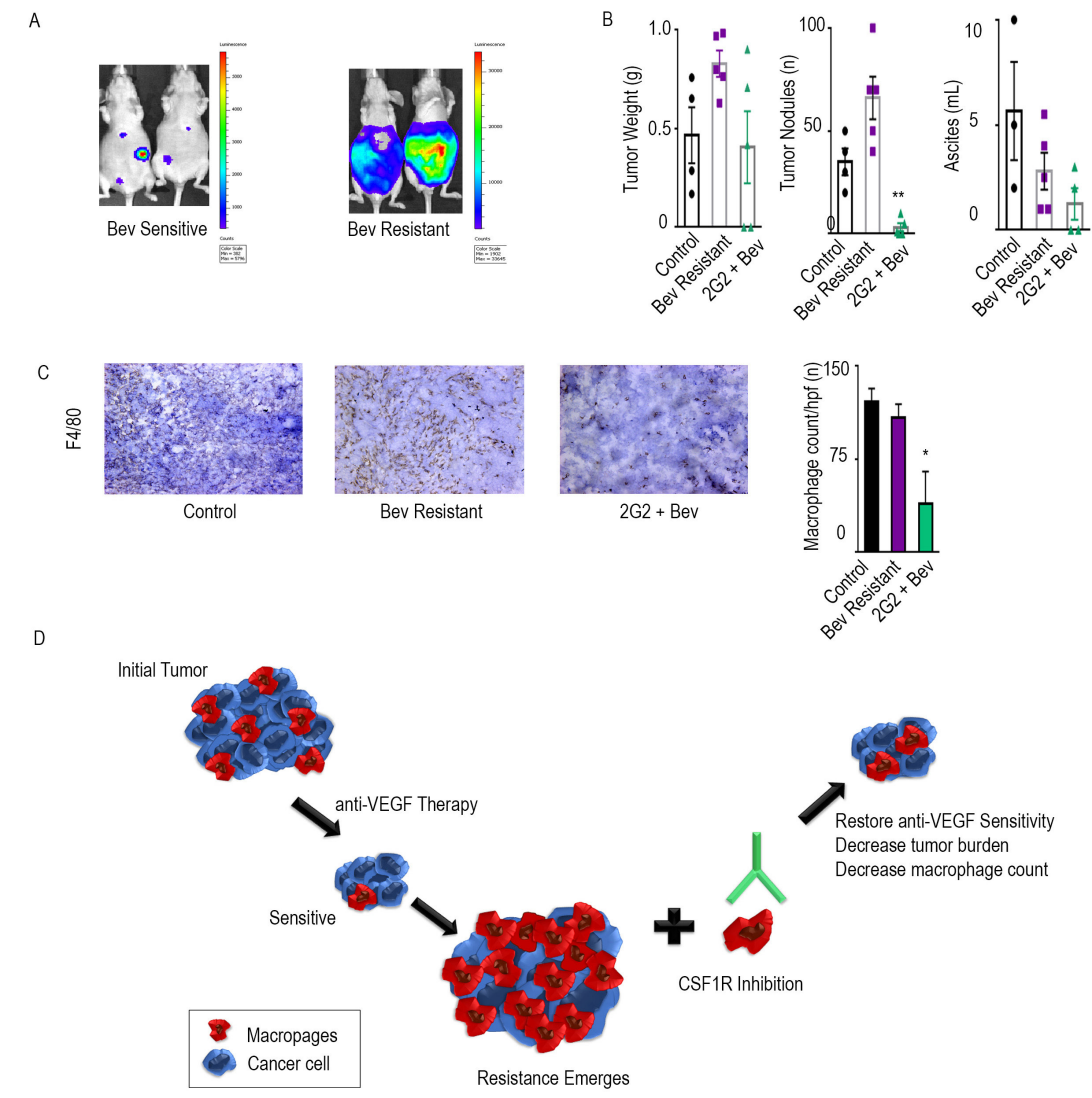
Additional CSF1R inhibitors have anti-tumor effects The adaptive resistance model was set up in the OVCAR5 ovarian cancer model in nude mice. **A.** represents the bioluminescence signal of mice responding and those resistant to bevacizumab after exposure of 1 minute. Tumor weight, tumor nodules, and volume of ascites for groups treated with bevacizumab and the combination of bevacizumab and 2G2 are shown in bar graphs **B.**. Macrophage counts of each group are represented with immunohistochemical images using F4/80 as a macrophage marker **C.**. The bar graphs represent the quantification of macrophages per group. Schematic representation of the model **D.**. * denotes p≤0.05 and ** denotes p≤0.01.

To test for consistency of effect, we also tested the efficacy of a CSF1R targeted mouse monoclonal antibody, 2G2, in combination with bevacizumab [7]. Nude mice were injected intraperitoneally with HGSC cell line, OVCAR5. Treatment groups were similar to those described previously, 1) control, 2) bevacizumab, and 3) 2G2, beginning on day 21. Mice were imaged *via* IVIS every week and then separated into sensitive and resistant to bevacizumab, based on bioluminescence imaging (Figure 4A). Consistent with the experiments described above, the addition of 2G2 to anti-VEGF therapy at the point of bevacizumab resistance improved outcomes in tumor burden, tumor nodules, and ascites (Figure 4B). Tumor weight was decreased by 51%, tumor nodules by 92%, and ascites by 55%. Macrophage count, as determined by F4/80 staining, was significantly decreased by 64% in the group that received 2G2 at the emergence of bevacizumab resistance, compared to the bevacizumab only group (Figure 4C).

## DISCUSSION

The key findings from our study are a decrease in tumor burden, ascites, and macrophage content when a CSF1R inhibitor is combined with anti-VEGF therapy in the setting of adaptive resistance. These effects were seen both with and without concurrent taxane chemotherapy. We show a change in expression of p-AKT, FAK, and PD-L1 markers in macrophages from tumor treated with CSF1R inhibition in the setting of adaptive resistance, as compared to samples that continued with only anti-VEGF therapy.

In patients with platinum-resistant and platinum-sensitive HGSC with relapsed disease, adding bevacizumab to chemotherapy significantly improves progression-free survival (and overall survival for platinum-sensitive patients) [2, 8]. These findings led to the FDA-approval of bevacizumab in both groups of patients. Despite improvement in survival, the effect is transient and while it often results in tumor shrinkage or stasis, tumor growth occurs after several months [3]. Although the exact mechanisms of resistance to anti-angiogenic drugs are not fully known, upregulation of pro-angiogenic factors, augmented pericyte coverage, and recruitment of bone-marrow derived pro-angiogenic cells are some of the components that have been shown to play a role [3, 9]. Our area of interest has been to study the role of bone-marrow derived pro-angiogenic cells in adaptive resistance to anti-angiogenesis drugs. Previous work in our lab showed a significant increase in macrophages in the tumor microenvironment with the emergence of adaptive resistance to anti-VEGF therapy in ovarian cancer mouse models. Several options for directly or indirectly targeting macrophages have been tested in preclinical mouse models or clinical trials, including CSF1R inhibitors, CCL2-CCR2 antibodies, bisphosphonates, trabectedin, CD40 agonists, IL-10 antibodies, CXCL12-CXCR4 axis drugs, and TLR7 agonists [10, 11]. All of these drugs have varying degree of side-effects, some more severe than others, such as osteonecrosis of the jaw with bisphosphonates, transaminitis with trabectidin, and back pain and spinal cord compression seen with CCL2 antibodies [12-14]. We chose to target the CSF1R pathway given the reasonably low side-effect profile seen in clinical trials with emactuzumab as well as its profound effect on macrophage proliferation survival, as described below.

CSF-1 and its receptor CSF1R have been studied as therapeutic targets because of their overexpression in HGSC and role in progression of disease. CSF1R is the primary receptor responsible for the survival, proliferation, and differentiation of macrophages, making it an ideal candidate to target TAMs in ovarian cancer. Tumor-associated macrophages promote cancer progression in most solid tumors, including ovarian cancer. The density of TAMs is highest in HGSC compared to other ovarian cancer histological subtypes and correlates with pathologic grade, having highest infiltration in grade 3 cancers [15].

Here, we present that CSF1R inhibition aids in overcoming adaptive resistance to anti-VEGF therapy, and we show pathways affected by macrophage depletion in the setting of adaptive resistance. We showed that CSF1R inhibitors not only deplete macrophages, but also significantly decrease tumor burden when combined with anti-VEGF therapy in multiple models of ovarian cancer. Adaptive resistance to anti-VEGF therapy is a substantial problem in patients with HGSC, leading to relapse in most cases [3]. In attempts to address this setback, we utilized a mouse model of adaptive resistance to anti-VEGF therapy and added in CSF1R inhibitor at the point of maximum resistance. Finally, we demonstrate possible pathways by which CSF1R inhibition restores sensitivity to anti-VEGF therapy. Based on our CyTOF results, we found pathways involving PD-L1, FAK, and p-AKT could be important for restoring sensitivity to anti-VEGF therapy.

## MATERIALS AND METHODS

### Cell lines

IG10 ovarian cancer cells were maintained in DMEM supplemented with 5% fetal bovine serum, 1x insulin-transferrin-sodium selenite supplement (Roche Diagnostics, Indianapolis, IN), and 0.1% gentamicin sulfate (Gemini Bioproducts, Calabasas, CA). OVCAR432 cell lines were maintained in RPMI 1640 supplemented with 15% fetal bovine serum and 0.1% gentamicin sulfate. OVCAR5 cell line was maintained in DMEM supplemented with 10% fetal bovine serum and 0.1% gentamicin sulfate. All cell lines were screened for mycoplasma and experiments were performed at 60-80% confluence.

### CSF1R inhibiting drugs

AC708 (aka PLX73086), a small molecule CSF1R inhibitor with significant specificity for CSF1R over PDGFRα/β, FLT3, and KIT [6] is currently being tested in cancer patients (NCT02673736). 2G2, the second inhibitor, is a chimeric murine IgG1 antagonistic antibody with high affinity for mouse CSF1R with a K_D_ of 0.2nM [7].

### Animal studies

Experiments involving human and murine cell lines were performed on 8-12 week old female athymic nude and 4-6 week old female C57/Bl6 mice, respectively, obtained from Taconic Farms (Hudson, NY). All experiments were done in accordance to protocols approved by MD Anderson Institutional Animal Care and Use Committee.

Tumor cells were injected intraperitoneally (1x 10^6^ cells/mouse for IG10, OVCAR432, and OVCAR5) into mice in all groups on day 0. The patient-derived cell line MDA-HGSC-1 (2414) was injected intraperitoneally as ascites, after being grown in NOD-SCID mice. Nude mice were treated with anti-VEGF antibody, bevacizumab, 5 mg/kg, twice weekly, intraperitoneal injection, while C57/Bl6 mice were treated with murine monoclonal VEGF-A and VEGFR-2 antibody, B20, 5 mg/kg, twice weekly, intraperitoneal injections (Genentech Inc, San Francisco, CA). AC708 was given 90 mg/kg, daily oral gavage. 2G2 was administered once weekly *via* intraperitoneal injection at a dose of 30 mg/kg. Paclitaxel 4 mg/kg once weekly, intraperitoneal injection was given to nude mice. Treatment for therapy experiments began 7 days after injection.

### Adaptive resistance model

IG10 and OVCAR432/OVCAR5 cells were transduced with lenti-virus encoding luciferase and inoculated intraperitoneally into C57/Bl6 and nude mice, respectively. Mice were treated with anti-VEGF therapy alone or anti-VEGF therapy and paclitaxel starting at approximately 21 days after inoculation and at that time imaged once weekly for luminescence signals using the Xeongen IVIS system. Mice were separated into “sensitive” and “resistant” to bevacizumab/B20 based on resolution or increase in disease burden documented by bioluminescence imaging. At the emergence of resistance, AC708 or 2G2 was added to anti-VEGF treatment.

### PDX model

Fresh tumor tissue obtained from surgical specimens was immediately processed for both storage and propagation in mice. NOD-SCID mice (The Jackson Laboratory) were used for all PDX model experiments. After processing, either 3x3 mm chunks of tumor tissue or ascites from patient-derived high-grade serous line, MDA-HGSC-1 (2414), were bilaterally implanted subcutaneously or injected intraperitoneally, respectively, into NOD-SCID mice. Once moribund, tumor from these mice was extracted, processed, and frozen down for storage, as well as implanted into more NOD-SCID mice for future experiments.

### CyTOF antibody conjugation

The following protocol was performed as noted in Han, et al [16]. Antibodies were either purchased preconjugated from DVS Sciences (Sunnyvale, CA), Biolegend (San Diego, CA), or conjugated at our institution *via* the following method. Using MaxPar Antibody Labeling Kit (DVS Sciences), purified, carrier-free antibody was conjugated with lanthanide isotopes. NanoDrop 2000 (Thermo Fisher Scientific) was used to determine protein concentration where absorbance of 1 at 280 nM equaled 1 mg/mL. To determine the metal contents of the conjugated antibody by CyTOF in solution, Claritas PPT Grade Multi-Element Solution 1 (SPEX CertiPrep, Metuchen, NJ) was used at 0.5 ppb as a standard.

### Mass cytometry staining

Tissue was harvested from mice inoculated with tumor, as stated above, and put in PBS on ice. Digestion cocktail of 0.375% collagenase Type I (Thermo Fisher), 250 units/mL DNAse I (Qiagen), and media was prepared. Samples were sliced into 1 mm fragments on ice and then incubated with digestion cocktail for 30 minutes in a 37°C water bath. Tumor samples were transferred to a 70 μm strainer and mechanically crushed, rinsed with PBS, and centrifuged. Lymphocyte separation media (Corning Life Sciences, Corning, NY) was added and samples were centrifuged. The interface was harvested and sample was washed with 0.5% BSA in PBS and resuspended in the same buffer. Viability staining was performed with 25 μM cisplatin at room temperature for 1 minute. Cells were washed with buffer and then blocked with Fc block (1:100, BD Sciences) for 5 minutes at 4°C. Metal-conjugated antibodies against surface markers were stained in 50-μL final volume for 30 minutes at room temperature. Cells were washed twice with buffer and then fixed with Foxp3 fixation buffer (eBioscience) at room temperature for 30 minutes. Foxp3 permeabilization buffer (eBioscience) was added and cells were washed twice. Cells were stained with intracellular markers in a 50-μL final reaction volume for 30 minutes at room temperature. After staining, cells were washed again with permeabilization buffer twice and stained with 0.5 mL of 1:1000 Iridium intercalator (Cat 201192A; DVS Sciences, Toronto, ON, Canada) diluted in PBS with 1.6% PFA for 30 minutes at room temperature. Cells were washed with buffer and resuspended in 0.1% BSA in MilliQ water. Samples were analyzed on a CyTOF mass cytometer using an AS5 Autosampler (both, DVS Sciences); 50 uL Eu151/153 calibration beads (Cat. 201073; DVS Sciences) were used in each sample to routinely normalize the raw CyTOF data before analysis. Data was saved in FCS3.0 format and analyzed with Flowjo.

### Immunohistochemistry/Immunofluorescence

Frozen sections were used for all mouse tissue. The sections were fixed in acetone and acetone-chloroform. After endogenous peroxide block using hydrogen peroxide in PBS and 3 washes of PBS, slides were incubated in primary antibody (F4/80, 1:100, Serotec) overnight at 4°C. Matching secondary antibodies (Jackson Immuno Research) were used at room temperature for 1 hour. Staining was developed using DAB for immunohistochemical staining with hematoxylin as the nuclear stain. Hoechst was used for nuclear staining for immunofluorescence.

### Statistical analysis

Differences in continuous variables were compared using Student’s *t* test or analysis of variance. We considered p ≤ 0.05 to be significant, demarcated as *, p ≤ 0.01 as **, p ≤ 0.001 as ***, and p ≤ 0.0001 noted as ****.

## SUPPLEMENTARY MATERIALS FIGURES AND TABLE


