# Supplementary material for: Macrophage depletion through colony stimulating factor 1 receptor pathway blockade overcomes adaptive resistance to anti-VEGF therapy
Source: Oncotarget. 2017 Aug 24;8(57):96496–505. doi: 10.18632/oncotarget.20410 (PMC5722500; doi:10.18632/oncotarget.20410)
Supplement: Supplementary file 1 [file oncotarget-08-96496-s001.pdf]

Macrophage depletion through colony stimulating factor 1 receptor pathway blockade overcomes adaptive resistance to anti-VEGF therapy

SUPPLEMENTARY MATERIALS

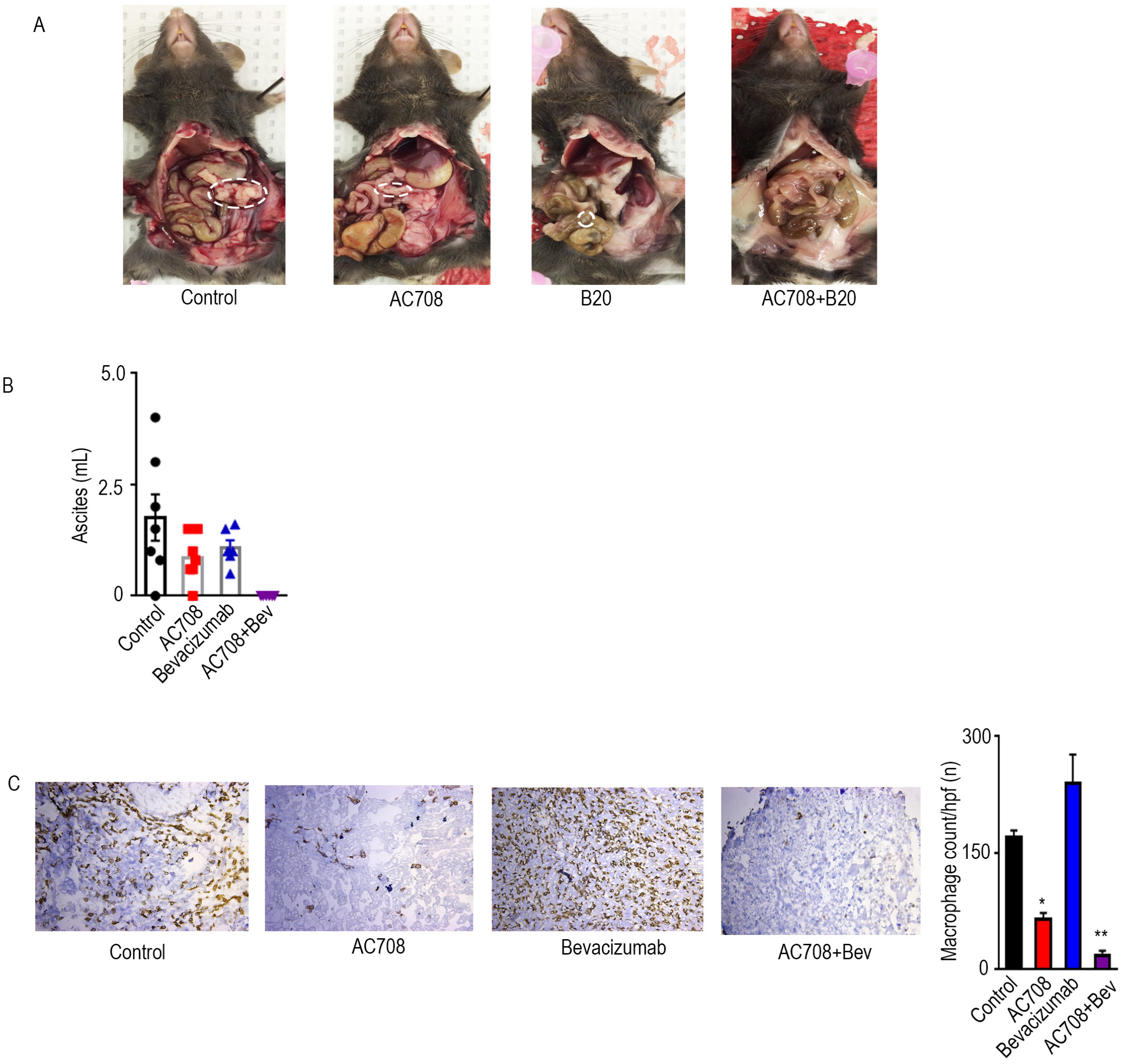

Supplementary Figure 1. ***AC708 in combination with B20 has anti-tumor effects.*** At time of dissection, representative pictures of mice from each group (no treatment, AC708, B20, combination) were taken **A.** Volume of ascites from PDX model is shown in **B.** Macrophage count by F4/80 antibody on immunohistochemistry was compared between groups for the PDX model **C.** The graph represents mean number of macrophages from 5 randomly selected high power fields at 20x high power. * denotes $p \leq 0.05$ and ** denotes $p \leq 0.01$.

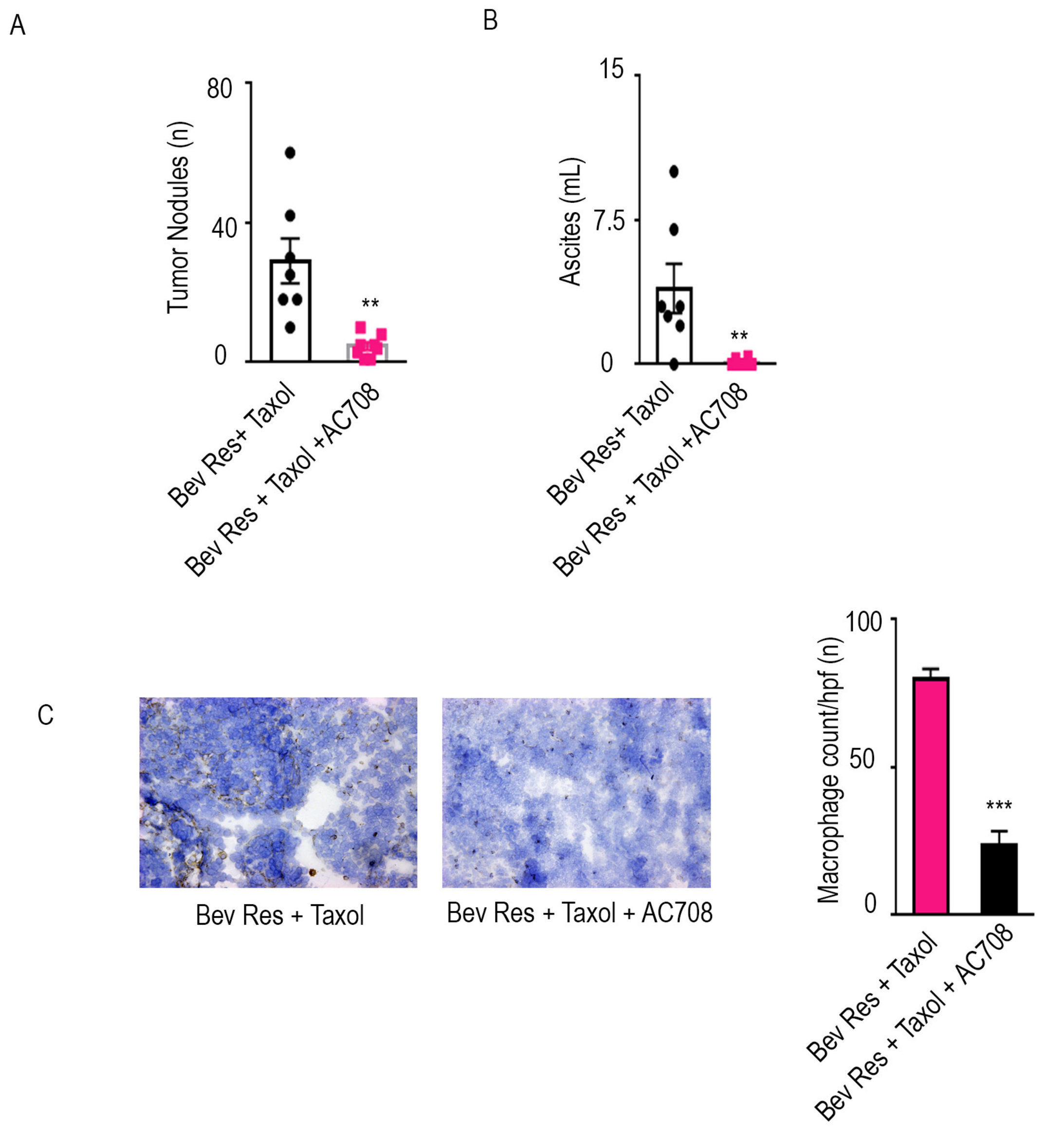

Supplementary Figure 2. ***AC708 has anti-tumor effects when combined with bevacizumab in the setting of adaptive resistance.*** **A.-B.** Number of tumor nodules and volume of ascites seen in OVCAR432 ovarian cancer model treated with bevacizumab, paclitaxel, and AC708 in the setting of adaptive resistance to bevacizumab. The corresponding tumors were stained with F4/80 antibody using immunohistochemistry and macrophages were quantified and are represented in the bar graph **C.** ** denotes $p \leq 0.01$ and *** denotes $p \leq 0.001$.

Supplementary Table 1: Markers for CyTOF Analysis.

No.	Isotope	Mass Channel	Myeloid panel		Surface / Intracellular	T-cell panel	Surface / Intracellular
1	139La	139	OX40L	S			
2	141Pr	141	FAK	I		Bcl-xL	I
3	142Nd	142	CD11c	S		clv-Caspase 3	I
4	143Nd	143					
5	144Nd	144	p-AKT	S		IL-2	I
6	145Nd	145	CD80	S			
7	146Nd	146				BIM	I
8	147Sm	147	CD45	S		CD45	S
9	148Nd	148	CD11b	S		Ki-67	I
10	149Sm	149	CD19	S			
11	150Nd	150	CD25	S		CD25	S
12	151Eu	151				CD28	S
13	152Sm	152	CD40	S		Bcl-6	I
14	153Eu	153				OX-40	S
15	154Sm	154	GM-CSF	I		4-1BB	S
16	155Gd	155				ICOS	S
17	156Gd	156	CD34	S		CD69	S
18	158Gd	158				Foxp3	I
19	159Tb	159	F4/80	S			
20	160Gd	160	PD-L2	S		p-PI3K	I
21	161Dy	161	IL-12	I		PD-1	S
22	162Dy	162	TNFa	I		TNFa	I
23	163Dy	163				CTLA-4	S
24	164Dy	164	PD-L1	S		TCRγδ	S
25	165Ho	165	HIF-1a	I		IFNg	I
26	166Er	166	CD206	S		Tim 3	S
27	167Er	167	IL-6	I			
28	168Er	168	CD8a	S		CD8a	S
29	169Tm	169					S
30	170Er	170	NK1.1	S		CD27	S
31	171Yb	171				CD44	S
32	172Yb	172	CD4	S		CD4	S
33	173Yb	173	Ly6G	S		LAG-3	S
34	174Yb	174	MHC Class II	S		IL17A	I
35	175Lu	175	Ly6C	S			
36	176Yb	176	B220	S			

Immune profiling was performed via CyTOF using a panel of T-cell and myeloid surface and intracellular markers. The isotope and mass channel for each marker is listed in the table. All markers are either listed as S for surface marker, or I for intracellular marker.
